# Exogenous marker-engineered mesenchymal stem cells detect cancer and metastases in a simple blood assay

**DOI:** 10.1186/s13287-015-0151-9

**Published:** 2015-09-21

**Authors:** Linan Liu, Shirley X. Zhang, Rangoli Aeran, Wenbin Liao, Mengrou Lu, George Polovin, Egest J. Pone, Weian Zhao

**Affiliations:** Department of Pharmaceutical Sciences, University of California, Irvine, 845 Health Sciences Road, Irvine, CA 92697 USA; Department of Biomedical Engineering, University of California, Irvine, 845 Health Sciences Road, Irvine, CA 92697 USA; Sue and Bill Gross Stem Cell Research Center, University of California, Irvine, 845 Health Sciences Road, Irvine, CA 92697 USA; Chao Family Comprehensive Cancer Center, University of California, Irvine, 845 Health Sciences Road, Irvine, CA 92697 USA; Edwards Lifesciences Center for Advanced Cardiovascular Technology, University of California, Irvine, 845 Health Sciences Road, Irvine, CA 92697 USA; Department of Biological Sciences, California State University, Long Beach, 1250 Bellflower Boulevard, Long Beach, CA 90840 USA

## Abstract

**Introduction:**

Mesenchymal stem cells (MSCs) are adult multipotent stem cells that possess regenerative and immunomodulatory properties. They have been widely investigated as therapeutic agents for a variety of disease conditions, including tissue repair, inflammation, autoimmunity, and organ transplantation. Importantly, systemically infused MSCs selectively home to primary and metastatic tumors, though the molecular mechanisms of tumor tropism of MSCs remain incompletely understood. We have exploited the active and selective MSCs homing to cancer microenvironments to develop a rapid and selective blood test for the presence of cancer.

**Methods:**

We tested the concept of using transplanted MSCs as the basis for a simple cancer blood test. MSCs were engineered to express humanized *Gaussia* luciferase (hGluc). In a minimally invasive fashion, hGluc secreted by MSCs into circulation as a reporter for cancer presence, was assayed to probe whether MSCs co-localize with and persist in cancerous tissue.

**Results:**

*In vitro*, hGluc secreted by engineered MSCs was detected stably over a period of days in the presence of serum. *In vivo* imaging showed that MSCs homed to breast cancer lung metastases and persisted longer in tumor-bearing mice than in tumor-free mice (*P* < 0.05). hGluc activity in blood of tumor-bearing mice was significantly higher than in their tumor-free counterparts (*P* < 0.05).

**Conclusions:**

Both *in vitro* and *in vivo* data show that MSCs expressing hGluc can identify and report small tumors or metastases in a simple blood test format. Our novel and simple stem cell-based blood test can potentially be used to screen, detect, and monitor cancer and metastasis at early stages and during treatment.

**Electronic supplementary material:**

The online version of this article (doi:10.1186/s13287-015-0151-9) contains supplementary material, which is available to authorized users.

## Introduction

Cancer is a leading cause of human morbidity and mortality, and its origins, biomarkers, and detection remain difficult to pinpoint [1]. Although early detection has proven to be a useful and often necessary first step to effectively manage and treat cancer [2], it remains a challenge at early stages to identify cancer, especially small tumors and metastases which account for over 90 % of cancer mortality [3, 4]. Methods of cancer detection based on imaging are non-invasive, but common drawbacks include high cost, low specificity or resolution, and the use of potentially irritating contrast agents [2]. For instance, positron emission tomography (PET), computed tomography (CT), and their combinations (PET-CT) are widely used for identifying and staging tumors but require high doses of ionizing radiation and have limited specificity and resolution [5]. Other imaging modalities, such as magnetic resonance imaging (MRI) and ultrasound, do not use radiation but are still unable to achieve spatial resolution smaller than several millimeters [6, 7]. On the other hand, tissue biopsies are invasive and suffer from false negatives for heterogeneous tumors, and obtaining biopsies from multiple small disseminated tumors (e.g., metastases) is impractical. Cancer screening also uses tests for biomarkers, including circulating tumor cells, exosomes, proteins, and nucleic acids. Recently, scientists have developed nanoparticle-based synthetic biomarkers composed of mass-encoded peptides that can be released upon tumor protease cleavage and then detected in urine [8, 9]. Such approaches, however, still rely on passive delivery of nanoparticles to tumors via the enhanced permeability and retention (EPR) effect and on limited types of endogenous proteins, both of which are cancer type-specific. More recently, scientists have also reported a probiotic microbe-based system to deliver synthetic biomarker for cancer detection in urine [10]. Nevertheless, cancer biomarker discovery has led to only a few biomarkers used in clinical diagnosis since cancer biomarkers frequently suffer from low sensitivity and specificity [11].

In particular, cancer heterogeneity and evolution make it challenging to rely on molecular biomarkers for cancer detection [1]. For example, the commonly used cancer biomarkers prostate-specific antigen for prostate cancer and *BRCA1/2* gene mutations for breast cancer can identify only about 25 % and 10 % to 25 % of the patients in each cancer type, respectively [12]. Indeed, it has been widely accepted that a single biomarker typically lacks the sensitivity and specificity that are necessary for useful diagnosis. Intriguingly, recent research indicates that most cancers are caused by stochastic events rather than predictable mutations [13]. Thus, finding biomarkers that recognize multiple types of cancers with no common genetic basis is likely less promising than previously thought. In summary, there is clearly an unmet clinical need for sensitive early-stage cancer and metastasis tests that can “universally” identify many types of cancers independently of specific biomarkers from healthy controls and other conditions that share similar symptoms (e.g., inflammation) as well as to discriminate different (sub)types of cancers at different stages.

Cells, including immune and stem cells, act as autonomous and adaptive agents and these properties have recently been used for cancer treatment and drug delivery [14–17]. In particular, mesenchymal stem (or stromal) cells (MSCs) have been tested as therapeutic agents because of their intrinsic regenerative and immunomodulatory features [18–23]. MSCs are under investigation for treating a wide array of diseases, including diabetes, myocardial infarction, stroke, and autoimmune diseases [24–26]. MSCs are also the world’s first manufactured stem cell product to receive clinical approval (i.e., Prochymal manufactured by Osiris (Columbia, MD, USA) was approved in Canada to treat graft-versus-host disease) [26], suggesting that they may be a safe source for diagnostic and therapeutic uses in humans. Importantly, systemically infused MSCs preferentially home to and integrate with tumors, including both primary tumors and metastases in different anatomical locations [24]. As we have recently reviewed [22], mounting evidence now suggests that MSCs possess leukocyte-like, active homing mechanisms for tumor tropism involving a variety of adhesion molecules (e.g., P-selectin and vascular cell adhesion molecule-1) and tumor-derived cytokines, chemokines, and growth factors (e.g., CXCL12 and platelet-derived growth factor). This selective and active homing ability makes MSCs appealing vectors for localized delivery of therapeutics to treat cancers, including gliomas, melanomas, breast cancer, and lung metastases, in ongoing clinical trials [15, 24]. In addition, MSCs engineered with probes (such as luciferase) have been used to detect and image tumors *in situ* [19, 27]. However, imaging methods such as PET/single-photon emission computed tomography and MRI, which are currently used for cell tracking after infusion are limited by the same aforementioned disadvantages of cancer detection [2].

**Scheme 1:**
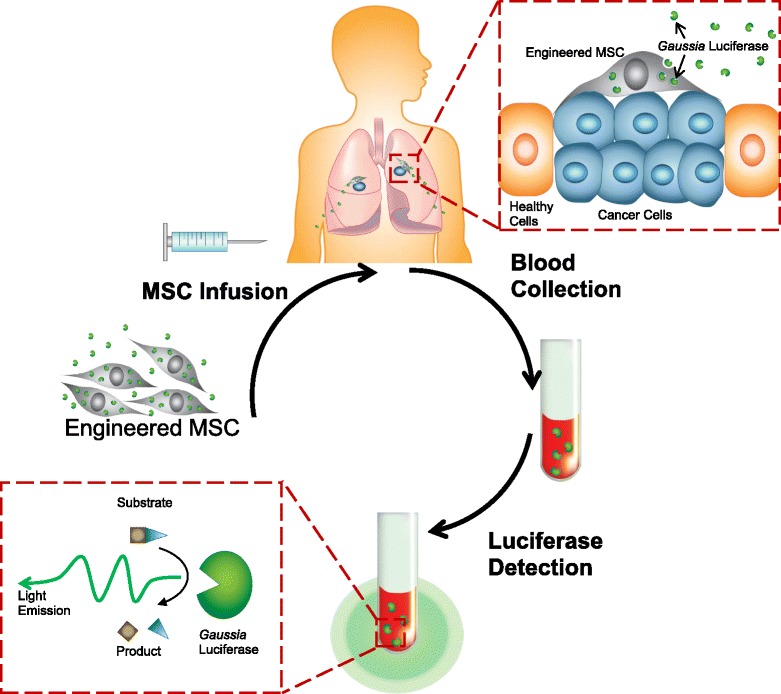
Using engineered mesenchymal stem cells (MSCs) to detect cancer. Engineered MSCs (*gray*) secreting humanized *Gaussia* luciferase (hGluc) (*green*) are systemically administered into patients with cancer (breast cancer lung metastasis in this case). Engineered MSCs home to tumor (*cyan*) niche and persist, secreting hGluc into blood. Then patient blood can be collected and hGluc activity measured

In this article, we present the concept of using exogenous MSCs as the basis for a simple cancer blood test (Scheme 1). Here, we hypothesize that, owing to their tumor tropism property, MSCs engineered with a secreted reporter can actively and specifically home to tumor sites regardless of the type and location of the tumors and persist there longer compared with MSCs in healthy microenvironments. MSCs engineered to express humanized *Gaussia* luciferase (hGluc) [28–31] were systemically administered to mice harboring breast cancer cells, exhibited tumor tropism and persistence, and secreted hGluc into the bloodstream of tumor-bearing mice. Thus, MSCs engineered with secreted reporters can potentially be developed into a blood test for broad cancer screening and monitoring.

## Methods

### Cell lines and cell culture

Human bone marrow MSCs were obtained from the Texas A&M Health Science Center and were expanded to within passages 3–6. The cells were routinely maintained in minimum essential medium alpha (MEMα) (Life Technologies, Carlsbad, CA, USA) supplemented with 15 % fetal bovine serum (FBS) (Atlanta Biologicals, Norcross, GA, USA) and 1 % penicillin-streptomycin (PenStrep) (100 U/ml; Life Technologies) at 37 °C in a humidified incubator containing 5 % CO_2_. The human breast cancer cell line MDA-MB-231 was obtained from American Type Culture Collection (ATCC) (Manassas, VA, USA). These cells were grown in Leibovitz’s L-15 medium containing L-glutamine (Corning, Corning, NY, USA) and supplemented with 10 % FBS and 1 U/ml PenStrep at 37 °C in a humidified incubator without CO_2_. The human colon cancer cell line LoVo was obtained from ATCC. These cells were grown in Kaighn’s Modification of Ham’s F-12 Medium (F-12 K; ATCC) and supplemented with 10 % FBS and 1 U/ml PenStrep at 37 °C in a humidified incubator with 5 % CO_2_. The 293 T-LV cell line (GenTarget, San Diego, CA, USA) was cultured in Dulbecco’s modified Eagle’s medium (DMEM) (Life Technologies) supplemented with 15 % FBS, non-essential amino acid (NEAA) (1X, 100 U/ml; Life Technologies), and 1 U/ml PenStrep at 37 °C in a humidified incubator containing 5 % CO_2_. All the cell experiments and procedures were performed after the approval from the University of California, Irvine (UCI) Institutional Biosafety Committee (protocol number 2012–1412).

### Generation of lentiviral vectors

The following lentiviral (LV) vectors were used in this study: LV-eGFP, LV-Fluc-tdT, and LV-hGluc. The sequences of interest from pUCBB-eGFP (#32548; Addgene, Cambridge, MA, USA), pcDNA3.1(+)/Luc2=tdT (#32904; Addgene), and pSV40-Gluc (New England BioLabs, Ipswich, MA, USA) were cloned into the promoterless LV transfer vector LV-PL4 (GenTarget).

### Lentiviral transduction

All LV constructs were packaged (pMD2.G, #12259; pRSV-Rev, #12253; pMDLg/pRRE, #12251; all from Addgene) as LV vectors in 293 T-LV cells [32] by using Lipofectamine LTX and PLUS™ Reagents (Life Technologies). MSCs and breast cancer cells were transduced with LVs by incubating virions in a culture medium containing 100 μg/ml protamine sulfate (Sigma-Aldrich, St. Louis, MO, USA). After selection with medium containing 10 μg/ml Puromycin (MP Biomedicals, Santa Ana, CA, USA), cells were visualized for fluorescent protein expression by using fluorescence microscopy.

### *In vitro* bioluminescence assays

LV-Fluc-tdT MSCs (Fluc-tdT-MSCs) expressing firefly luciferase (Fluc) or LV-hGluc MSCs (hGluc-MSCs) expressing humanized *Gaussia* luciferase (hGluc) were seeded in serially diluted concentrations. After the cells were washed with PBS (Lonza, Basel, Switzerland), luciferase substrates (150 μg/ml D-luciferin for Fluc, PerkinElmer, Waltham, MA, USA, or 20 μM coelenterazine (CTZ) for hGluc, NanoLight Technologies, Pinetop, AZ, USA) were added and the activities of Fluc and hGluc were imaged as previously described [33]. Conditioned medium (CM) of hGluc-MSCs was harvested and filtered. CM (5 μl) was then mixed with human serum (Atlanta Biologicals) with or without PBS dilution to final serum concentrations of 0 %, 5 %, 50 %, or 100 %, incubated at 37 °C at various times as indicated, and hGluc activity was measured with 20 μM CTZ (final concentration in a final volume of 200 μl). Mouse blood was collected as described [34] and added into ¼ volume of EDTA (Sigma-Aldrich) solution (50 mM, pH=8.0). Blood (5 μl) was mixed with 100 μl of 100 μM CTZ, and hGluc activity was measured immediately. All bioluminescent assays were performed with an IVIS Lumina (Caliper LifeSciences, Hopkinton, MA, USA) or a plate reader (BioTek, Winooski, VT, USA). All samples above were measured in triplicate.

### Cell implantation and imaging *in vivo*

LV-Fluc-tdT MDA-MB-231 (Fluc-tdT-231) or LV-eGFP MDA-MB-231 (eGFP-231) breast cancer cells or LoVo colon cancer cells (0.5×10^6^ ; 2.5×10^6^/ml in DPBS) were implanted intravenously (i.v.) into nonobese diabetic/severe combined immunodeficiency gamma (NSG) mice (5 weeks, #005557; The Jackson Laboratory, Bar Harbor, ME, USA). Five weeks later, *in vivo* Fluc activity from Fluc-tdT-231 cells was measured as described [35]. Briefly, *in vivo* Fluc signal was imaged with IVIS Lumina 10 minutes after intraperitoneal (i.p.) injection of D-luciferin (150 mg/kg in DPBS; Lonza) into mice. hGluc-MSCs or Fluc-tdT-MSCs (10^6^; 5×10^6^/ml in DPBS) were systemically infused into the mice harboring breast cancer cells and into healthy control mice. hGluc-MSCs were labeled with the Dil lipophilic dye (5 μl/10^6^ cells; Life Technologies) by incubation at 37 °C for 20 minutes before infusion. Mice were anesthetized with 2~3 % of isoflurane (Western Medical Supply, Arcadia, CA, USA), and *in vivo* Fluc activity was measured at the indicated time points. Imaging was performed with the IVIS Lumina (n=4 in each case). All animal experiments and procedures were performed after the approval from the UCI Institution of Animal Care and Use Committee (protocol number 2012–3062) and conducted according to the Animal Welfare Assurance (#A3416.01).

### Tissue processing and immunohistochemistry

Tissues were collected and flash frozen in Tissue-Tek O.C.T™ Compound (Sakura Finetek, Torrance, CA, USA), with or without overnight fixation in 4 % paraformaldehyde (Amresco, Solon, OH, USA), and with overnight incubation in 30 % sucrose solution (Amresco). Sections 8 μm thick were taken by cryostat and stained following an immunohistochemistry protocol for eGFP (sheep polyclonal IgG; Pierce Biotechnology, Rockford, IL, USA) and Fluc (rabbit polyclonal IgG; Abcam, Cambridge, UK). Briefly, slides were fixed in acetone (Thermo Fisher Scientific, Waltham, MA, USA) at −20 °C for 10 minutes, permeabilized in 0.1 % Triton X-100 (Sigma-Aldrich) for 10 minutes, and blocked in 0.1 % Triton X-100 with 5 % normal donkey serum (Sigma-Aldrich) for 30 minutes. Primary antibodies were diluted 1:100 from the stock solution in 0.05 % Tween-20 (Sigma-Aldrich) in PBS and applied overnight at 4 °C. Slides were washed in 1X PBS, and then secondary antibodies (donkey anti-sheep IgG conjugated to Alexa Fluor 488, donkey anti-rabbit IgG conjugated to Alexa Fluor 594, Jackson ImmunoResearch Laboratories, West Grove, PA, USA) were diluted 1:500 from the stock solution in 0.05 % Tween-20 in PBS and applied for 30 minutes at room temperature. TOTO-3 Iodide (2.4 μM; Life Technologies) was added to the secondary antibody incubation. DAPI (4*'*,6-diamidino-2-phenylindole) (50 μg/ml; Life Technologies) in PBS was applied to slides for 10 minutes before mounting. Slides were washed in PBS and mounted with DPX (Di-N-butyle phthalate in xylene) (Sigma-Aldrich) or Fluoromount-G (SouthernBiotech, Birmingham, AL, USA).

### Statistical analysis

Data were analyzed by Student’s *t* test when comparing two groups and by analysis of variance when comparing more than two groups. Data were expressed as mean ± standard deviation or as mean ± standard error of the mean, and differences were considered significant at *P* values of less than 0.05.

## Results

### Humanized *Gaussia* luciferase is secreted from engineered MSCs *in vitro* and is stable and detectable in blood

**Fig. 1:**
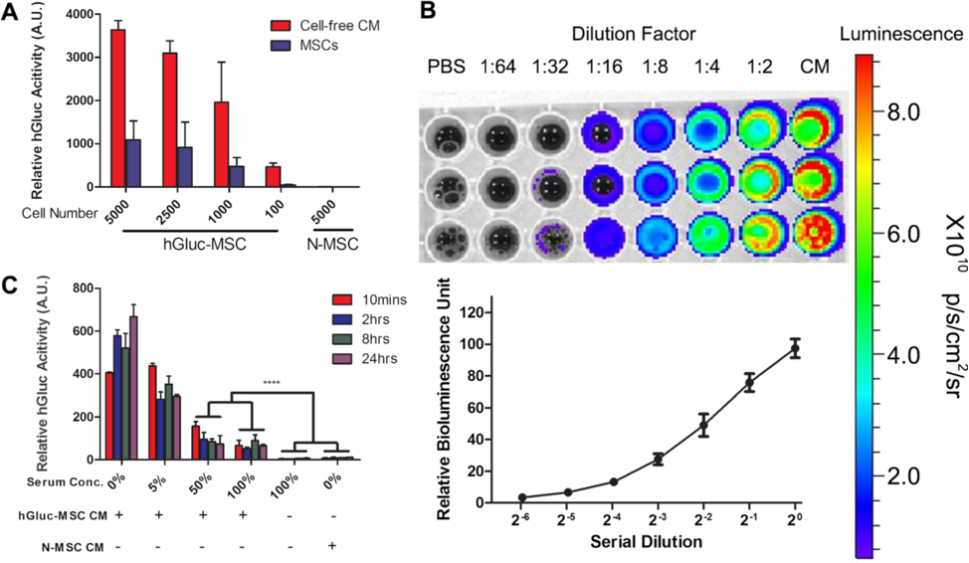
Humanized *Gaussia* luciferase (hGluc) is secreted *in vitro* and is stable in blood. **a** Mesenchymal stem cells expressing humanized *Gaussia* luciferase (hGluc-MSC) and native MSCs (N-MSCs) were seeded onto 96-well plates. Twenty-four hours later, cell-free conditioned medium (CM) was harvested. The hGluc substrate coelenterazine (CTZ) was added with a final concentration of 20 μM. hGluc activity was measured immediately by using a plate reader (absorbance at wavelengths of 300-700 nm, exposure time = 2 s). **b** Serial dilution of hGluc-MSC CM was performed in PBS, and CTZ was added at a final concentration of 20 μM. hGluc activity was measured with an IVIS Lumina (exposure time = 0.5 s). Color scale: minimum = 6.64×10^8^, maximum = 8.93×10^9^. **c** CM of hGluc-MSCs was harvested and incubated with human serum for 10 minutes and 2, 8, or 24 hours at 37 °C. A final concentration of 20 μM of CTZ was added, and hGluc activity was measured immediately (exposure time = 2 s). hGluc activity was detectable in 100 % serum. ****P < 0.0001. Error bar: mean ± standard deviation. *A.U.* arbitrary units, *PBS* phosphate-buffered saline

Human bone marrow MSCs were stably transduced with lentivirus to express secreted humanized *Gaussia* luciferase (hGluc) as described above. To determine whether hGluc is secreted in an active form by MSC, cell-free CM was harvested from hGluc-MSCs 24 hours after MSC seeding at different concentrations (100, 1000, 2500, or 5000 cells per cm^2^). The substrate CTZ was added and hGluc activity was measured for both cells and CM (Fig. 1a). hGluc activity increased with increasing cell number (Fig. 1a). In addition, hGluc activity in CM was 3- to 6-fold higher than inside cells (Fig. 1a), indicating that hGluc expressed by engineered MSCs is secreted in active form, as expected. hGluc-MSC CM was serially diluted with PBS and hGluc activity was measured *in vitro* and found to exhibit a linear function of concentration, in agreement with earlier reports [33, 36, 37] (Fig. 1b). To demonstrate whether luciferase activity from hGluc-MSCs is detectable and sufficiently stable in blood, human serum either directly (100 %) or serially diluted in PBS was mixed with hGluc-MSCs CM. hGluc activity remained detectable (*P* < 0.0001) after 24 hours co-incubation and was not decreased significantly over time (Fig. 1c), indicating that hGluc-MSCs can be a stable marker in blood assays *in vitro*. Finally, since both firefly luciferase (Fluc-tdT) and hGluc would be used *in vivo* (below), any potential cross-reactivity between Fluc-tdT and hGluc-MSCs was measured (Additional file 1: Figure S1). These two luciferases were substrate-specific and no cross-reaction was observed, as reported. Overall, these data show that hGluc expressed by engineered MSCs is secreted *in vitro*, is stable in human serum for up to 24 hours, and exhibits substrate-specific enzyme activity.

### Engineered MSCs home to tumor sites and persist longer in the lungs of the tumor-bearing mice

**Fig. 2:**
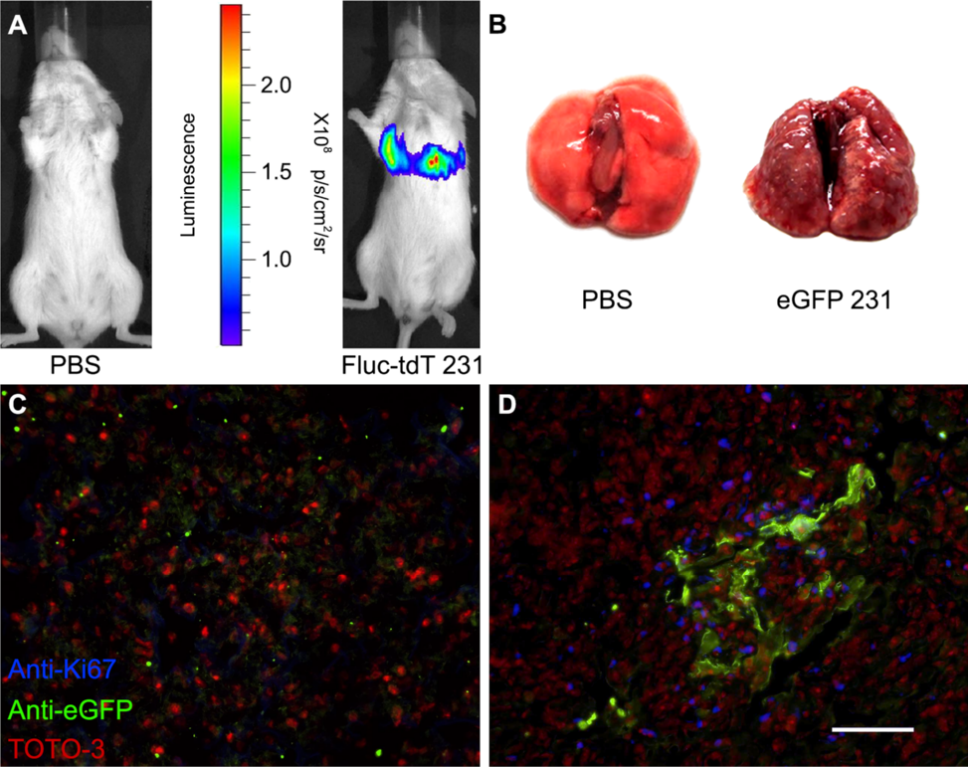
Human-derived breast cancer was observed in xenotransplantation murine model. **a** Five weeks after 0.5×10^6^ Fluc-tdT-231 were seeded i.v., NSG mice were injected intraperitoneally with D-Luciferin (150 mg/kg in Dulbecco’s PBS) and *in vivo* Fluc activity was measured with IVIS Lumina 10 minutes after substrate administration. Exposure time = 5 s. Color scale: minimum = 5.13×10^7^, maximum = 2.46×10^8^. **b** Representative pictures of tumor-free (*left*) and tumor-bearing (*right*) lungs. Eight weeks after MDA-MB-231 breast cancer cells or PBS were seeded i.v., NSG mice were euthanized and lungs were harvested. Frozen sections of lungs of **c** tumor-free mice and **d** eGFP-231 tumor-bearing mice sacrificed 5 weeks after cancer seeding were stained with anti-eGFP (*green*), anti-Ki67 (*blue*), and TOTO-3 (*red*). Scale bar: 50 μm. *eGFP* enhanced green fluorescent protein, *i.v.* intravenously, *NSG* nonobese diabetic/severe combined immunodeficiency gamma, *PBS* phosphate-buffered saline

As MSCs are reported to naturally home to tumor sites [18, 19], we tested this phenomenon in our experiment as a preliminary step to using MSCs that secrete hGluc as a diagnostic tool for cancer detection and localization. Human breast cancer-derived MDA-MB 231 cells were labeled with eGFP or Fluc-tdT and implanted intravenously (i.v.) into immunodeficient NSG mice (Fig. 2) to establish a simple *in vivo* mouse model of breast cancer that has metastasized in the lungs [38, 39]. Tumor mass was observed in lungs both *in vivo* (Fig. 2a) and *ex vivo* (Fig. 2b, d), whereas no tumor-related signal was seen in healthy lungs (Fig. 2a, c). Owing to the fact that hGluc is secreted by MSCs and to its diluted and limited signal under whole animal imaging conditions with IVIS Lumina [40] (data not shown), we used MSCs engineered with intracellular Fluc-tdT [41] for real-time imaging and localization of MSCs in tumors *in situ.* Fluc-tdT-MSCs were simultaneously labeled with red fluorescent protein (RFP) to assess Fluc transduction efficiency and to image any co-localized MSCs and tumor cells in subsequent *ex vivo* immunohistochemistry. Both Fluc activity and RFP signal from Fluc-tdT-MSCs were observed *in vitro* (Additional file 2: Figure S2), demonstrating that engineered MSCs express Fluc (Additional file 2: Figure S2A) with high transduction efficiency (>90 % RFP^+^; Additional file 2: Figure S2B-D).

**Fig. 3:**
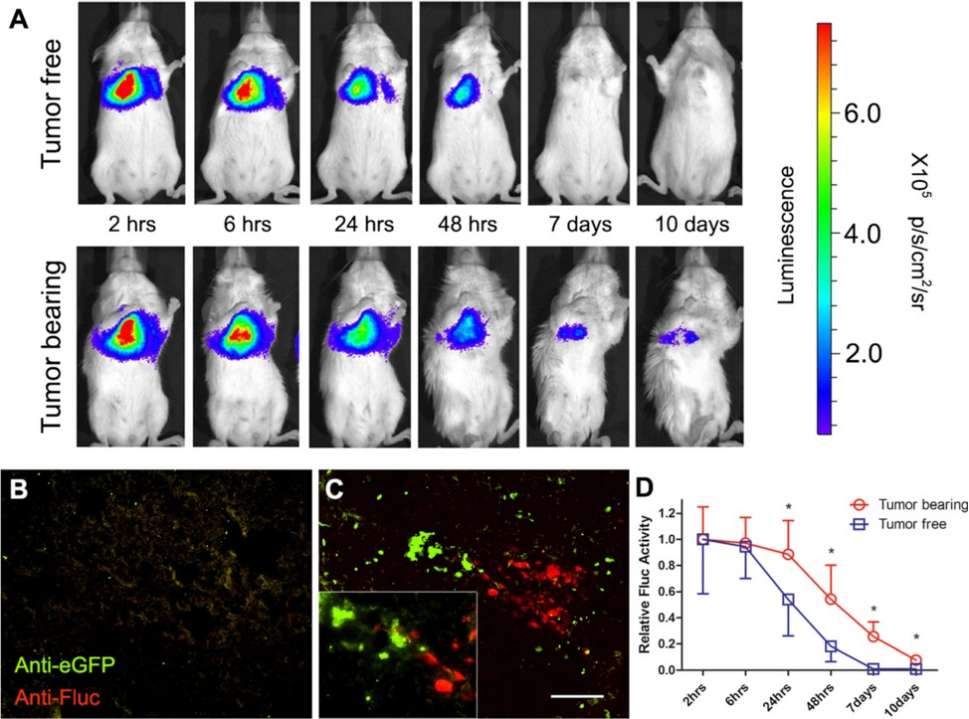
Mesenchymal stem cells home to tumor site and persist longer than in healthy mice. **a** Five weeks after eGFP-231 were seeded intravenously into NSG mice, 10^6^ Fluc-tdT-MSCs were administered systemically into both tumor-free (*top*) and tumor-bearing (*bottom*) mice. Then mice were injected intraperitoneally with D-Luciferin (150 mg/kg in Dulbecco’s phosphate-buffered saline), and *in vivo* Fluc activity was measured at different time points (2, 6, 24, and 48 hours and 7 and 10 days after MSC infusion) by using an IVIS Lumina to begin data acquisition 10 minutes after substrate administration (exposure time = 60 s; n=4 in each group). MSCs were cleared out faster in tumor-free mice. Color scale: minimum = 6.50×10^4^, maximum = 7.50×10^5^. Frozen sections of lungs of **b** tumor-free mice and **c** eGFP-231 tumor-bearing mice sacrificed 10 days after Fluc-tdT-MSC infusion were stained with anti-eGFP (*green*) and anti-Fluc (*red*) antibodies. MSCs were observed to home to tumor niche. Scale bar: 50 μm. **d** Fluc activity measured at different time points was quantified and normalized to the time point of 2 hours. Error bar: mean ± standard error of the mean. *P <0.05. n=4 in each group. *eGFP* enhanced green fluorescent protein, *Fluc* firefly luciferase, *MSC* mesenchymal stem cell, *NSG* nonobese diabetic/severe combined immunodeficiency gamma, *tdT* tdTomato red fluorescent protein

**Fig. 4:**
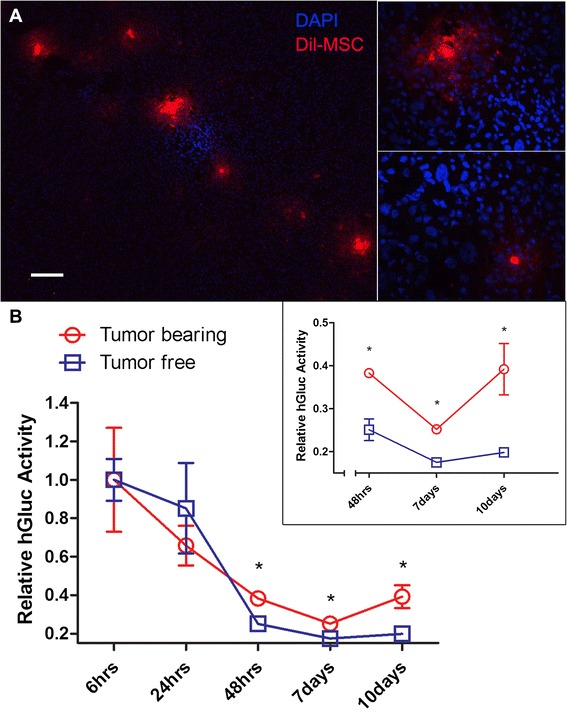
*Gaussia* luciferase (hGluc) is active in murine blood and the signal is elevated in tumor-bearing mice. **a** Frozen sections of lungs of tumor-bearing mice sacrificed 10 days after Dil-labeled hGluc-MSC administration were stained with DAPI and then imaged by fluorescence microscopy. MSCs (*red*) were observed to home to tumor niche (*dense blue*). Scale bar: 100 μm. **b** Five weeks after Fluc-tdT-231 were seeded intravenously into NSG mice, 10^6^ hGluc-MSCs were administered systemically into both tumor-free and tumor-bearing mice. Then murine blood was harvested and hGluc activity was measured at different time points (6, 24, and 48 hours and 7 and 10 days after MSC infusion) with IVIS Lumina immediately after substrate was added. hGluc activity measured at different time points was quantified and normalized to the time point of 6 hours. The inset graph shows that the hGluc activity in blood between tumor-bearing and tumor-free mice is significantly different from 48 hours after MSC infusion. Error bar: mean ± standard error of the mean. *P <0.05. Exposure time = 30 s. n=4 in each group. *DAPI* 4*'*,6-Diamidino-2-phenylindole, *MSC* mesenchymal stem cell

To investigate any differences in MSCs homing between cancer-bearing and healthy mice, 10^6^ Fluc-tdT-MSCs were systemically infused into mice with or without breast cancer. Mice were anesthetized and *in vivo* Fluc activity was measured after i.p. administration of D-luciferin substrate into mice at the indicated time points. *In vivo* imaging demonstrated that MSCs were detectable in tumor-bearing mice for as long as 10 days after systemic administration (Fig. 3a). *Ex vivo* immunohistochemistry data confirmed that engineered MSCs homed to the tumor niche *in vivo* (Figs. 3c and 4a). As we hypothesized, engineered MSCs persisted significantly longer in tumor-bearing lungs, especially at later time points (Fig. 3a). We then quantified the Fluc signal and found that significant differences between tumor-bearing and tumor-free mice emerged 24 hours after MSC infusion and lasted until 10 days after infusion (Fig. 3d, n=4, *P* < 0.05). To test whether our technology can be applied to other types of cancer, we investigated fused Fluc-tdT-MSCs into mice with lung metastasis of colon cancer. Similar results were observed (Additional file 3: Figure S3) which demonstrate engineered MSCs could home to and stay in tumor-bearing lungs for a significantly longer time compared with tumor-free lungs. Our data, along with mounting evidence of MSC tumor tropism in the literature [18, 22, 42, 43], suggest that the *in vivo* persistence of engineered MSCs in tumor-bearing compared with healthy animals provides a viable “marker” for broad cancer detection.

### hGluc secreted by engineered MSCs can be assayed in the blood of tumor-bearing mice

We next investigated whether MSCs that were engineered to express hGluc can be used to detect metastasis of breast cancer to the lungs. hGluc was chosen as the reporter in this study because of its high sensitivity, lack of nonspecific cross-reactivity to other substrates (e.g., Additional file 1: Figure S1), and linear signal over a wide concentration range (Fig. 1b). In addition, hGluc has a short half-life *in vivo* (20 minutes), allowing repeated real-time testing without undesirable excessive signal accumulation, but a long half-life *in vitro* (6 days), allowing convenient sample storage [33]. As hGluc is secreted, it cannot be used as a marker to co-localize MSCs and tumor as seen in Fig. 3c for intracellular Fluc. Therefore, in this set of experiments, we stained hGluc-MSCs with the Dil lipophilic dye before they were infused i.v. into mice. Like Fluc-tdT-MSCs, Dil-MSCs were detectable in the tumor niche up to 10 days post-infusion (Fig. 4a). Mouse blood was collected at the indicated time points, and hGluc activity was measured. Although the detected signal decayed rapidly over time as expected, the difference of hGluc activity in blood between tumor-bearing and tumor-free mice was significant starting from 48 hours after MSC administration and lasting until 10 days post-infusion (Fig. 4b), suggesting that systemically infused hGluc-MSC can be used for the potential development of a simple blood assay for cancer detection in this murine model. In summary, this set of data supports the feasibility of using engineered MSCs with secreted hGluc as a blood test for the presence of cancer.

## Discussion

Early detection of cancer, especially metastasis, is a necessary and often critical first step to effectively treat and eradiate cancer. Traditional imaging tools and molecular biomarker-based assays are typically complex, expensive, and/or invasive for routine screening for most cancers; most importantly, they frequently do not possess the sensitivity and specificity to identify heterogeneous cancers at early stages. In our study, we developed a stem cell-based detection system that can detect cancer, including metastases, by collecting small amounts of blood with a minimally invasive procedure. Our engineered MSCs could home to tumor sites and persist there for significantly longer durations compared with healthy mice. The signal derived from engineered stem cells lasted longer compared with current imaging tracers [5], and no repeat administration was needed. With one single administration, the presence of tumor could be monitored continuously through a prolonged period of time, making MSCs a convenient tool for real-time cancer detection. Compared with acellular systems (e.g., antibodies and nanoparticles), the natural interactions between MSCs and tumor involve complex adaptive sensing and responding systems that enable more efficient and specific reporting of cancer and metastases. This intrinsic biological property of tumor homing therefore potentially allows our stem cell approach to “universally” identify many cancers regardless of their origins, types, and anatomical sites. In addition, stem cell-based probe delivery circumvents many hurdles associated with passive delivery (i.e., by direct administration or polymeric nanoparticles via the EPR effect), including penetrating the endothelium and the increased pressure associated with tumors. In addition, the use of distinct, exogenous markers (hGluc in this article) as surrogate markers to detect and monitor cancer is more advantageous than endogenous markers because of the lack of unique cancer biomarkers. In our assay, a positive detection of hGluc (even with a small signal) would indicate the presence of cancer, which therefore helps to eliminate the need for sophisticated signal normalization over background as required in conventional cancer detection assays. Therefore, our simple, noninvasive stem cell-based blood test might be useful for routine cancer screening, detecting small tumors and metastases, and monitoring cancer progression and recurrence during the course of treatment.

Since MSCs possess not only tumor tropism but also tropism for bone marrow and sites of inflammation and injury [20, 23], it remains important to distinguish those conditions from cancer when using MSC-based methods to detect cancer. In addition, given high cancer heterogeneity, our next-generation systems aim to engineer MSCs with activatable, cancer type-specific probes to further increase the assay specificity. The long-term goal is to establish a panel of tests that can effectively discriminate between cancer (sub)types and stages and distinguish between cancer and other disorders that share similar symptoms, including inflammation and injury.

MSCs were chosen in our current (first-generation) system because they can be easily obtained from multiple adult tissues [44], including bone marrow and fat, therefore avoiding ethical concerns. MSCs are also relatively easy to expand in culture and can be readily engineered to express functional therapeutics or reporters [14, 23]. Importantly, the clinically approved Prochymal and hundreds of other ongoing clinical trials have demonstrated that allogeneic MSCs are generally safe for use in the human without harsh immunosuppressive regimens. Nonetheless, as MSCs themselves may participate in cancer progression or regression [22], further considerations are required. The interactions between MSCs and cancer remain incompletely understood [14, 22], with different reports indicating conflicting findings from endogenous and exogenous MSCs on cancer progression [22, 45, 46]. Thus, safety tests and optimizations will likely be required to better control the fate of our engineered MSCs after cancer detection, though no obvious MSC-mediated cancer growth was observed within our detection window (Additional file 4: Figure S4). To mitigate this potential issue, for example, a suicide gene [47] can be engineered into our MSC-based system so that after completion of the cancer detection test, the remaining engineered MSCs can be eliminated by using exogenously administered drugs. For example, inducible human caspase-9 (iC9), which can be activated by a bio-inert small-molecule drug, has been used as a safety switch in clinical trials of cell therapy with limited immunogenicity [48]. Another limitation of our study is that we used a relatively large tumor burden as our model to demonstrate our proof-of-concept because of its technical simplicity. In the future, we will evaluate our engineered stem cell approach to detect early-stage cancer and metastases when they are small by using cancer models with smaller tumor burden by either reducing the cell number administered or at the early stages of the beast cancer progression. These future experiments will allow us to determine the smallest tumor size we can detect with our technology. Furthermore, our system may be used as companion diagnostics combined with other treatments, for example, identifying certain patients and monitoring side effects. Finally, our cell-based blood assay may represent a new platform for monitoring the fate and functions of transplanted cells as well as for assessing the *in vivo* microenvironment where they reside.

## Conclusions

We demonstrate for the first time, to the best of our knowledge, a simple blood test for cancer detection. This test is based on the premise of exploiting the natural tumor-homing ability of MSCs to further engineer them to express a secreted luciferase with optimal biocompatibility and kinetic parameters. Similar to our current murine studies, these “reporter MSCs” could be developed to identify the presence of small tumors or metastases in humans that would otherwise be undetectable by existing imaging modalities. We hope this simple “off the shelf” allogeneic stem cell-based diagnostic test can be used to screen, detect, and monitor cancer on a routine basis.

## Note

This article is part of an ‘Emerging Investigators’ collection showcasing the work of early career investigators who have demonstrated growing leadership in the field of stem cells and regenerative medicine. Other articles in the series can be found online at http://stemcellres.com/series/emerginginvestigators.

## Box 1. About Weian Zhao

 Weian Zhao is an assistant professor at the Department of Pharmaceutical Sciences, University of California, Irvine. He completed his B.Sc. and M.Sc. in chemistry at Shandong University, where he studied polymer, surface, and colloidal chemistry. In 2008, he received his Ph.D. in chemistry at McMaster University, where he focused on the use of functional nucleic acid to structure gold nanoparticles to construct well-defined nanostructures and biosensors. He then completed a Human Frontier Science Program Postdoctoral Fellow at Harvard Medical School, Brigham and Women’s Hospital and MIT, where he learned stem cell trafficking, and cell engineering for diagnostics and therapeutics. His current research focuses on the development of novel molecular, nano-, and micro-engineered tools for stem cell therapy and regenerative medicine, diagnosis and *in vivo* imaging, and elucidating stem cell and cancer biology.

## Additional files

Additional file 1: Figure S1. Firefly and humanized *Gaussia* luciferases are substrate-specific and not cross-reactive. hGluc-MSCs, Fluc-tdT-MSCs (Fluc-MSCs), and N-MSCs were seeded in 96-well plate. The firefly luciferase substrate D-luciferin (final concentration = 150 μg/ml) or the humanized *Gaussia* luciferase substrate CTZ (final concentration = 20 μM) was added, and luciferase activity was measured with a plate reader. Error bar: mean ± standard deviation. Exposure time = 2 s. *A.U.* arbitrary units, *CTZ* coelenterazine, *Fluc* firefly luciferase, *hGluc* humanized *Gaussia* luciferase, *MSC* mesenchymal stem cell, *tdT* tdTomato red fluorescent protein. (PNG 37 kb) Additional file 2: Figure S2. Engineered mesenchymal stem cells (Fluc-tdT-MSCs) express firefly luciferase (Fluc) and red fluorescent protein (tdT). (A) Fluc-tdT-MSCs (Fluc-MSCs) were seeded onto 96-well plate, and 24 hours later D-luciferin was added at a final concentration of 150 μg/ml. Fluc activity was measured with a plate reader. Error bar: mean ± standard deviation. Exposure time = 2 s. ****P* <0.001. (B-D) Fluc-tdT-MSC were imaged by fluorescence microcopy 24 hours after seeding. Scale bar: 50 μm. *A.U.* arbitrary units, *MSC* mesenchymal stem cell, *tdT* tdTomato red fluorescent protein. (PNG 6051 kb) Additional file 3: Figure S3. Systemically infused MSCs persist in the lungs of the LoVo cancer cell-bearing mice. Five weeks after LoVo colon cancer cells were seeded intravenously into NSG mice, 10^6^ Fluc-tdT-MSCs were administered systemically into both tumor-free (*blue*) and tumor-bearing (*red*) mice. Then mice were injected intraperitoneally with D-Luciferin (150 mg/kg in Dulbecco’s phosphate-buffered saline), and *in vivo* Fluc activity was measured at different time points (6, 24, and 48 hours and 7 and 10 days after MSC infusion) by using an IVIS Lumina to begin data acquisition 10 minutes after substrate administration (exposure time = 60 s). Fluc activity measured at different time points was quantified. Similar to the results with MDA-MB-231 breast cancer cells, MSCs were cleared out faster in tumor-free mice, showing that the tumor tropism of MSCs is applicable to multiple types of cancers. Error bar: mean ± standard error of the mean. **P* <0.05. n=4 for tumor-bearing mice and n=3 for tumor-free mice. *Fluc* firefly luciferase, *MSC* mesenchymal stem cell, *NSG* nonobese diabetic/severe combined immunodeficiency gamma, *tdT* tdTomato red fluorescent protein. (PNG 128 kb) Additional file 4: Figure S4. Engineered mesenchymal stem cell (hGluc-MSC) infusion has no influence on the growth of cancer metastasis size *in vivo*. Five weeks after Fluc-tdT-231 were seeded intravenously into NSG mice, 10^6^ hGluc-MSCs or PBS was administered systemically into tumor-bearing mice (day 0). *In vivo* Fluc activity was measured with IVIS Lumina 10 minutes after substrate administration before (day 0) and 10 days after MSC infusion (day 10). Exposure time = 5 s. Relative metastasis index (RMI) = Luciferase activity on day 10 (after) / Luciferase activity on day 0 (before). N=4 for each group. *hGluc* humanized *Gaussia* luciferase, *MSC* mesenchymal stem cell, *n.s.* not significant, *NSG* nonobese diabetic/severe combined immunodeficiency gamma, *PBS* phosphate-buffered saline, *tdT* tdTomato red fluorescent protein. (PNG 126 kb)

## Abbreviations

ATCC: American Type Culture Collection
*BRCA1/2*: Breast cancer 1/2
CM: Conditioned medium
CT: Computed tomography
CTZ: Coelenterazine
CXCL12: C-X-C motif chemokine 12
DPBS: Dulbecco’s phosphate-buffered saline
EDTA: Ethylenediaminetetraacetic acid
eGFP: Enhanced green fluorescent protein
EPR: Enhanced permeability and retention
FBS: Fetal bovine serum
Fluc: Firefly luciferase
hGluc: Humanized *Gaussia* luciferase
i.p.: Intraperitoneal
i.v.: Intravenously
LV: Lentiviral
MRI: Magnetic resonance imaging
MSC: Mesenchymal stem cell
NSG: Nonobese diabetic/severe combined immunodeficiency gamma
PBS: Phosphate-buffered saline
PenStrep: penicillin-streptomycin
PET: Positron emission tomography
RFP: Red fluorescent protein
tdT: tdTomato red fluorescent protein
UCI: University of California, Irvine
