# Supplementary figures and images for: Exogenous marker-engineered mesenchymal stem cells detect cancer and metastases in a simple blood assay
Source: Stem Cell Res Ther. 2015 Sep 21;6(1):181. doi: 10.1186/s13287-015-0151-9 (PMC4578609; doi:10.1186/s13287-015-0151-9)

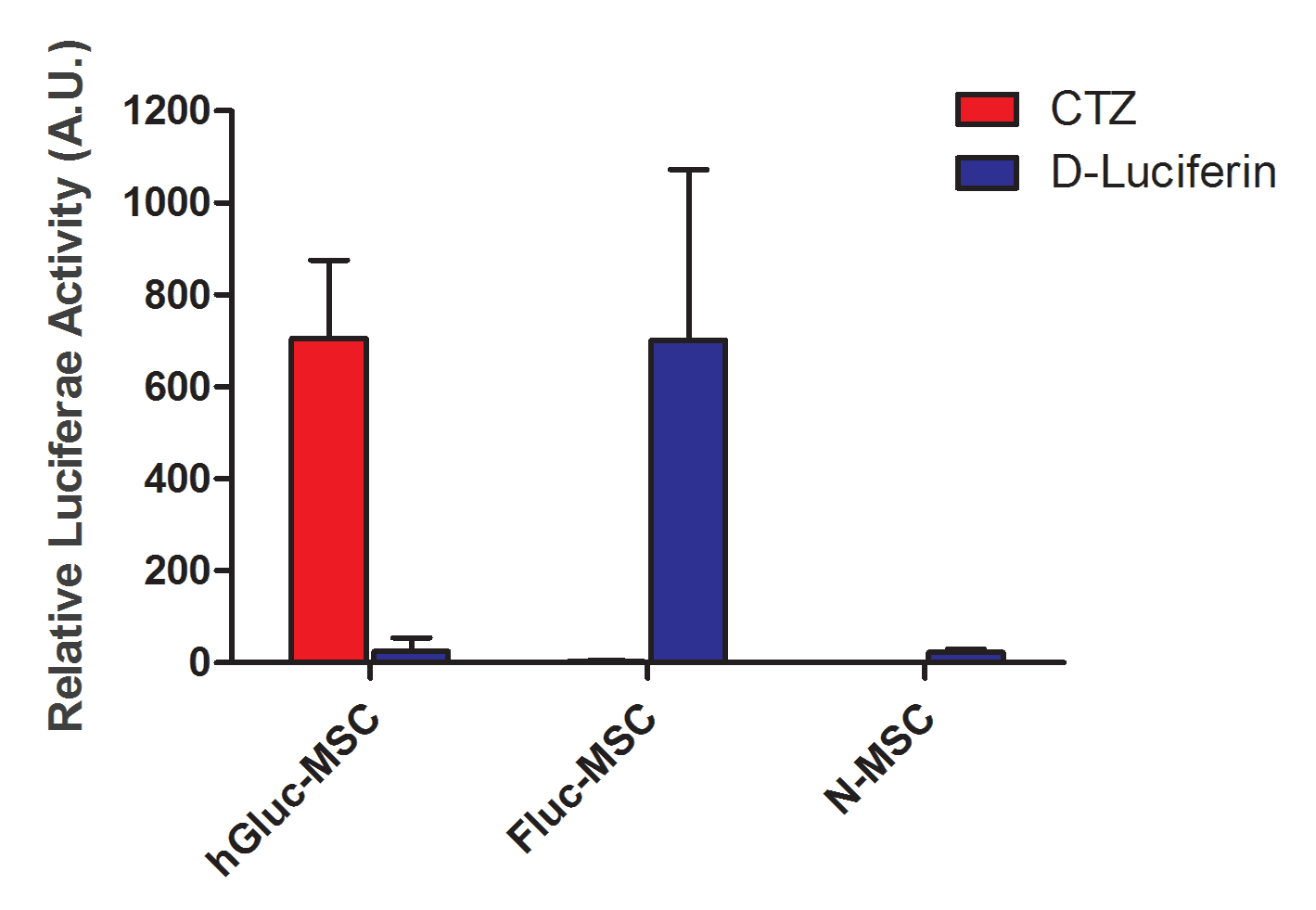

Supplement: Additional file 1: Figure S1. — Firefly and humanized Gaussia luciferases are substrate-specific and not cross-reactive. hGluc-MSCs, Fluc-tdT-MSCs (Fluc-MSCs), and N-MSCs were seeded in 96-well plate. The firefly luciferase substrate D-luciferin (final concentration = 150 μg/ml) or the humanized Gaussia luciferase substrate CTZ (final concentration = 20 μM) was added, and luciferase activity was measured with a plate reader. Error bar: mean ± standard deviation. Exposure time = 2 s. A.U. arbitrary units, CTZ coelenterazine, Fluc firefly luciferase, hGluc humanized Gaussia luciferase, MSC mesenchymal stem cell, tdT tdTomato red fluorescent protein. (PNG 37 kb) [file 13287_2015_151_MOESM1_ESM.png]

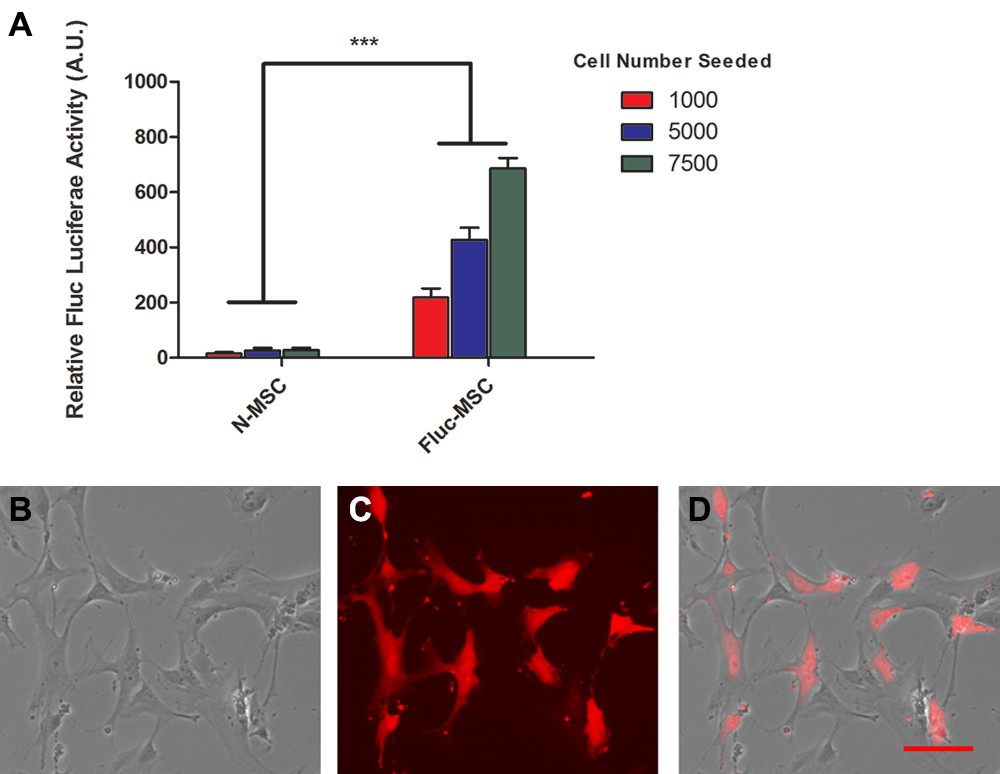

Supplement: Additional file 2: Figure S2. — Engineered mesenchymal stem cells (Fluc-tdT-MSCs) express firefly luciferase (Fluc) and red fluorescent protein (tdT). (A) Fluc-tdT-MSCs (Fluc-MSCs) were seeded onto 96-well plate, and 24 hours later D-luciferin was added at a final concentration of 150 μg/ml. Fluc activity was measured with a plate reader. Error bar: mean ± standard deviation. Exposure time = 2 s. ***P <0.001. (B-D) Fluc-tdT-MSC were imaged by fluorescence microcopy 24 hours after seeding. Scale bar: 50 μm. A.U. arbitrary units, MSC mesenchymal stem cell, tdT tdTomato red fluorescent protein. (PNG 6051 kb) [file 13287_2015_151_MOESM2_ESM.png]

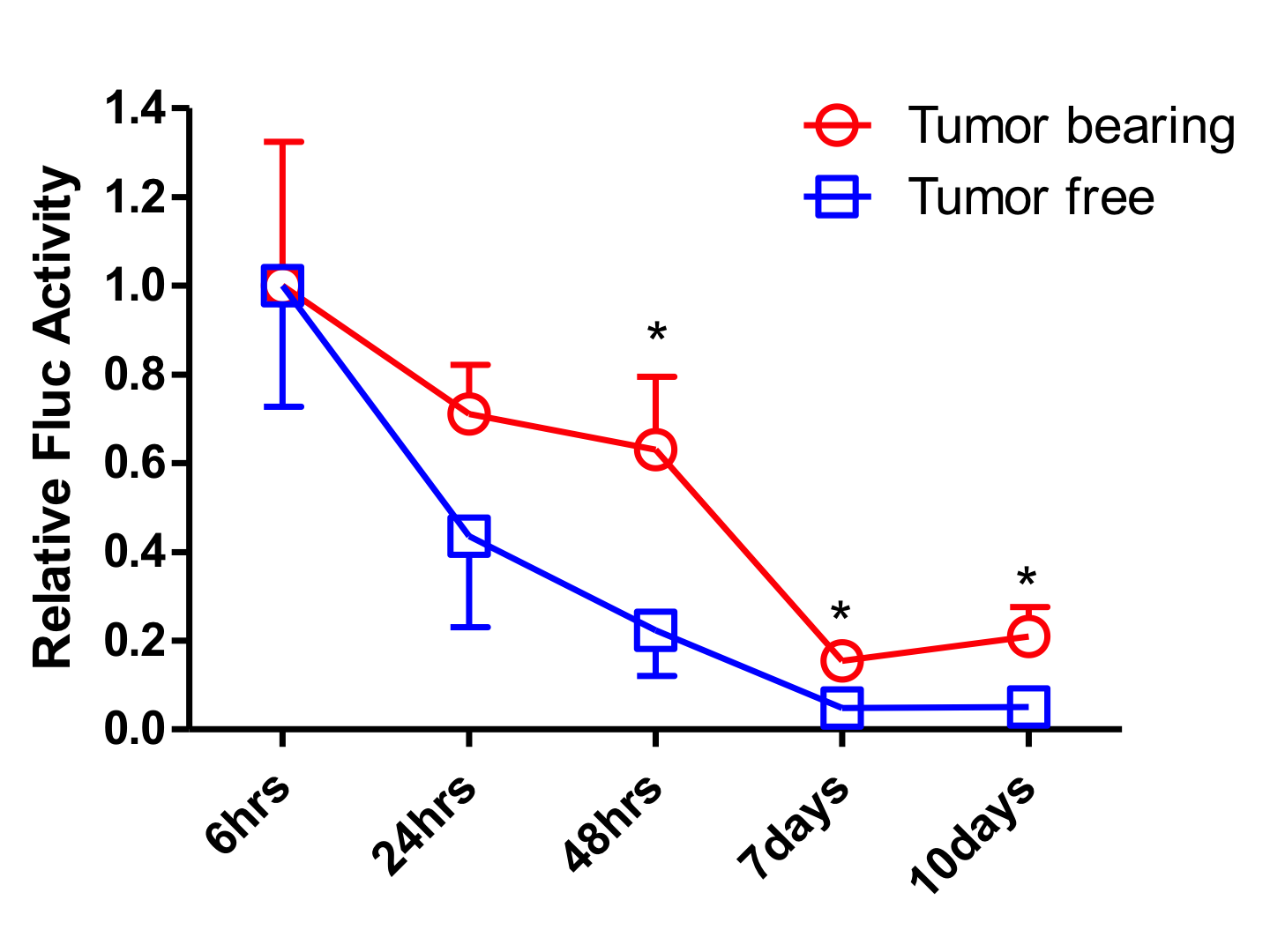

Supplement: Additional file 3: Figure S3. — Systemically infused MSCs persist in the lungs of the LoVo cancer cell-bearing mice. Five weeks after LoVo colon cancer cells were seeded intravenously into NSG mice, 106 Fluc-tdT-MSCs were administered systemically into both tumor-free (blue) and tumor-bearing (red) mice. Then mice were injected intraperitoneally with D-Luciferin (150 mg/kg in Dulbecco’s phosphate-buffered saline), and in vivo Fluc activity was measured at different time points (6, 24, and 48 hours and 7 and 10 days after MSC infusion) by using an IVIS Lumina to begin data acquisition 10 minutes after substrate administration (exposure time = 60 s). Fluc activity measured at different time points was quantified. Similar to the results with MDA-MB-231 breast cancer cells, MSCs were cleared out faster in tumor-free mice, showing that the tumor tropism of MSCs is applicable to multiple types of cancers. Error bar: mean ± standard error of the mean. *P <0.05. n=4 for tumor-bearing mice and n=3 for tumor-free mice. Fluc firefly luciferase, MSC mesenchymal stem cell, NSG nonobese diabetic/severe combined immunodeficiency gamma, tdT tdTomato red fluorescent protein. (PNG 128 kb) [file 13287_2015_151_MOESM3_ESM.png]

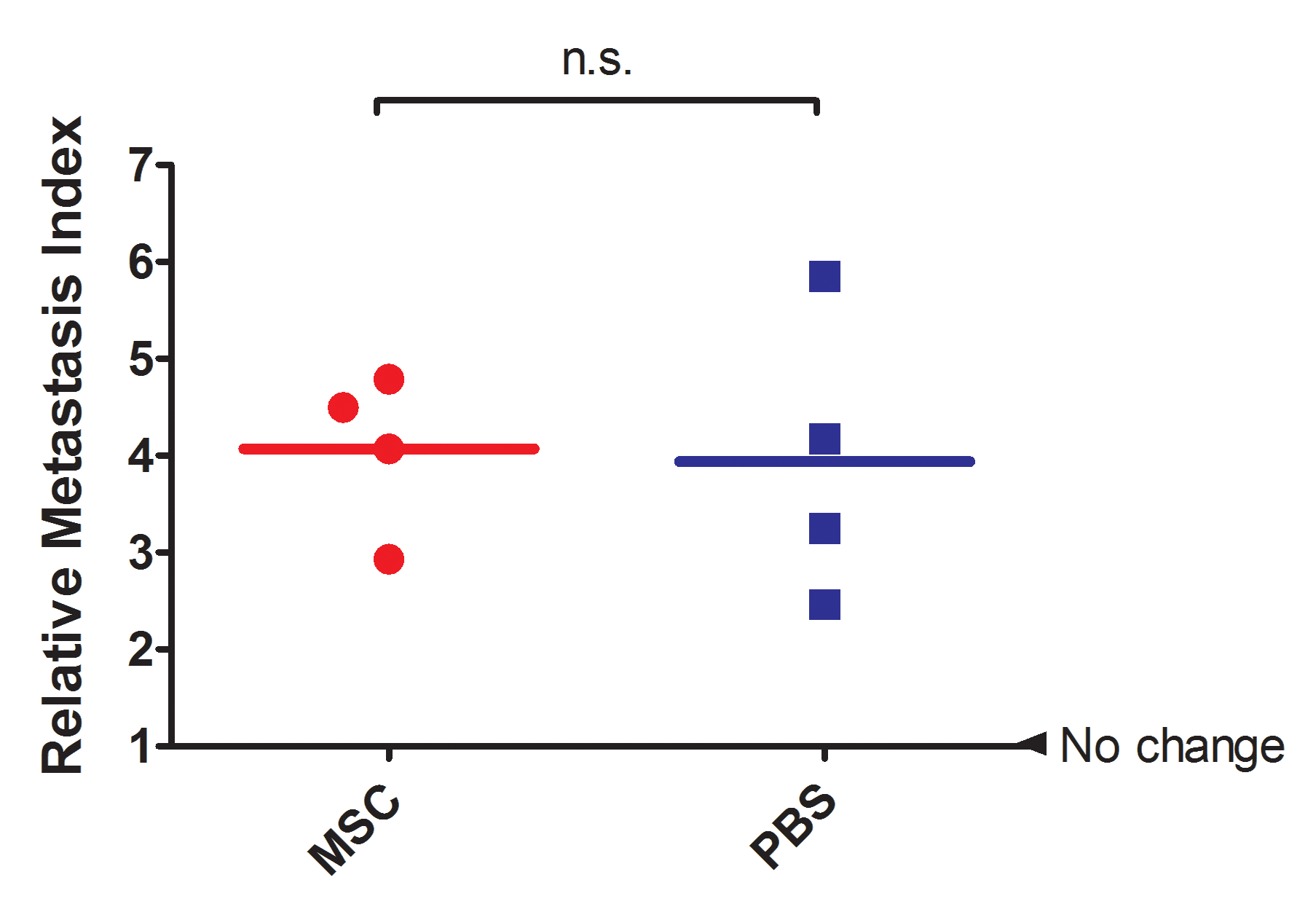

Supplement: Additional file 4: Figure S4. — Engineered mesenchymal stem cell (hGluc-MSC) infusion has no influence on the growth of cancer metastasis size in vivo. Five weeks after Fluc-tdT-231 were seeded intravenously into NSG mice, 106 hGluc-MSCs or PBS was administered systemically into tumor-bearing mice (day 0). In vivo Fluc activity was measured with IVIS Lumina 10 minutes after substrate administration before (day 0) and 10 days after MSC infusion (day 10). Exposure time = 5 s. Relative metastasis index (RMI) = Luciferase activity on day 10 (after) / Luciferase activity on day 0 (before). N=4 for each group. hGluc humanized Gaussia luciferase, MSC mesenchymal stem cell, n.s. not significant, NSG nonobese diabetic/severe combined immunodeficiency gamma, PBS phosphate-buffered saline, tdT tdTomato red fluorescent protein. (PNG 126 kb) [file 13287_2015_151_MOESM4_ESM.png]
